# Book Notices

**Published:** 1878-02-20

**Authors:** 


### BOOK NOTICES.

MALARIA AND STRUMA IN THEIR RELATION TO THE ETIOLOGY OF SKIN DISEASES; read before the American Dermatological Association at Niagara Falls, September, 1877.

We have read the above paper with great interest. It was prepared by Professor L. P. Yandell, of Louisville, and evinces on the part of the author an active and investigating mind.

Impressed by the interest and practical importance of the subject, we brought the matter to the notice of the Atlanta Medico-Chirurgical Association, and a brief of the discussion which it elicited may be seen in the present issue of our journal. From the novelty of the theory enunciated by Dr. Yandell, and the impromptu character of the remarks made, it would seem that our statement of the views of Dr. Yandell was to some extent misunderstood by certain of the disputants. Our statement of the case was in substance the same as that of the learned author himself, to-wit: "Malaria is the chief source of acute skin disease. Scrofula is the chief source of chronic skin disease. The more inveterate cases of skin disease are often due to the existence of these two things." The subject of the pamphlet we regard as important, and well worthy the attention of the profession.

THE DRUGGIST'S HAND BOOK OF PRIVATE FORMULAS. By John H. Nelson. Cleveland, Ohio, 1878.

The above is a neat little volume of about 200 pages, designed "to place before every druggist reliable formulas for preparing well known medicines not given in the U. S. Dispensatory. These preparations—elixirs, emulsions, medicated syrups, wines, etc., have become so universal that there is scarcely a druggist who does not have more or less sale for them, and to be enabled to prepare them is not only a source of satisfaction, but is also very desirable on account of the increase of profits." The book must prove useful, not only to druggists, but to physicians who are under the necessity of preparing their own medicines.

HIGHER MEDICAL EDUCATION: The True Interests of the Public and of the Profession. An Address introductory to the 112th Course of Lectures in the Medical Department of the University of Pennsylvania. October, 1877.

This is a very interesting address on the subject of medical education. The author takes high ground, and asserts "that the time has arrived when both the profession and the public are prepared to demand that reforms—yes, extensive reforms—shall be made in the American system of medical education."

MECHANISM OF JOINTS. By Harrison Allen, M.D., Professor of Comparative Anatomy and Zoology in the University of Pennsylvania, etc., extracted from the Transactions of the International Medical Congress. September, 1877.

A paper of considerable interest and practical importance.

### RECEIPTED.

N J Long, H Perdue, A W Mann, I H Goss, J C Wallace, John Riches, Jno Vandergriff, S W Eaton, J S Bacon, Joseph Underwood, W J Lee, L K Branch, J B Foster, S B Wall, D R Fox, W W Ward, H Allison, I H Wysong, G F Taylor, Preston Pond, J I Groover, John Slaughter, Lewis Hadden, J Dellworth, C F Rogers, M L Barron, T S Roach, B M Bledsoe, J W Unger, R H Lee, W T Kendall, W H Russell, W B Williamson, R E Hutchins, McCabe & Lanier, J E G Terrell, B R Doyle, J H Calfee, W H Calfee, T J Jones, W P Lawton, J R Johnson, S Y T Carter, W T Mathes, E H Hale, A H Sellers.

